# Cellular Interaction and Tumoral Penetration Properties of Cyclodextrin Nanoparticles on 3D Breast Tumor Model

**DOI:** 10.3390/nano8020067

**Published:** 2018-01-26

**Authors:** Gamze Varan, Viorica Patrulea, Gerrit Borchard, Erem Bilensoy

**Affiliations:** 1 Department of Nanotechnology and Nanomedicine, Hacettepe University, 06800 Ankara, Turkey; isikgamze@gmail.com; 2 School of Pharmaceutical Sciences, University of Geneva-University of Lausanne, 1211 Geneva, Switzerland; viorica.patrulea@unige.ch (V.P.); Gerrit.Borchard@unige.ch (G.B.); 3 Department of Pharmaceutical Technology, Faculty of Pharmacy, Hacettepe University, 06100 Ankara, Turkey

**Keywords:** 3D spheroid, amphiphilic cyclodextrin, cancer, cell culture, nanoparticles, Paclitaxel

## Abstract

Amphiphilic cyclodextrins are biocompatible oligosaccharides that can be used for drug delivery especially for the delivery of drugs with solubility problems thanks to their unique molecular structures. In this paper, Paclitaxel was used as a model anticancer drug to determine the inclusion complex properties of amphiphilic cyclodextrins with different surface charge. Paclitaxel-loaded cyclodextrin nanoparticles were characterized in terms of mean particle diameter, zeta potential, encapsulation efficacy, drug release profile and cell culture studies. It was determined that the nanoparticles prepared from the inclusion complex according to characterization studies have a longer release profile than the conventionally prepared nanoparticles. In order to mimic the tumor microenvironment, breast cancer cells and healthy fibroblast cells were used in 3-dimensional (3D) cell culture studies. It was determined that the activities of nanoparticles prepared by conventional methods behave differently in 2-dimensional (2D) and 3D cell cultures. In addition, it was observed that the nanoparticles prepared from the inclusion complex have a stronger anti-tumoral activity in the 3D multicellular tumor model than the drug solution. Furthermore, polycationic amphiphilic cyclodextrin nanoparticles can diffuse and penetrate through multilayer cells in a 3D tumor model, which is crucial for an eventual antitumor effect.

## 1. Introduction

Breast cancer is the most common cancer in women comprising 30% among all types of cancer [1]. Successful breast cancer treatment depends on efficient and safe delivery of chemotherapeutic agents to the tumor site. Conventional chemotherapy is administered mainly through intravenous (IV) infusion and therefore the anticancer drugs need to be in soluble form during this injection/infusion. Like most anticancer agents, Paclitaxel (PCX), which is the clinical first choice in breast cancer therapy, is practically insoluble in water. It therefore needs to be formulated with co-solvents like Cremophor EL^®^, which is causing severe side effects as a result of rapid crystallization of the very lipophilic drug upon dilution during iv infusion [2]. 

The use of solubilizers was avoided with the United States Food and Drug Adminisration (FDA) approval of albumin nanoparticle bound PCX (Abraxane^®^) in 2005 for breast cancer treatment at a significantly lower dose [3]. In a different formulation strategy, amphiphilic cyclodextrin nanoparticles were reported to protect PCX from recrystallization in aqueous dispersion thereby improving its safety, as cytotoxicity and hemolysis studies previously performed by our group showed [4,5].

Cyclodextrins (CDs) are natural oligomers, which are enzymatic degradation products of starch. CDs are macrocyclic oligosaccharides composed of α(1,4)-linked glucopyranose subunits [6]. Natural CDs are named according to the number of glucopyranose subunits. The widely used natural CDs are α-CD, β-CD and γ-CD consisting of six, seven or eight glucopyranose units, respectively. CDs are potential candidates as drug carrier systems because of their unique physical and chemical properties. They are typically torus shaped, having a hydrophobic inner cavity and a hydrophilic surface. CDs allow for the encapsulating of poorly soluble anticancer drugs in their hydrophobic inner cavity and mask the physicochemical properties of the guest molecules [7]. However, a major drawback of CDs is hemolysis and nephrotoxicity upon injection [8,9,10]. In order to overcome this disadvantage of natural CDs and to add self-assembling properties to this multifunctional excipient, amphiphilic CDs have been synthesized in recent years [11]. 

Amphiphilic CDs are cyclic oligosaccharide derivatives designed by grafting hydrocarbon chains onto the hydroxyl groups of either the primary and/or the secondary face of natural CDs [12]. Their unique structure gives them the ability to form nanoparticles spontaneously and to include active molecules in their hydrophobic inner cavity as well as within their long aliphatic chains [13]. Major advantages of amphiphilic CDs are the improved interaction with biological membranes, enhancement of inclusion complex capacity, prevention of CD-induced hemolysis and spontaneous formation of nanospheres or nanocapsules avoiding the use of surfactants or other surface active agents [4].

Nanoparticles are drug delivery systems that are capable of passively targeting cancer cells. Owing to their size in the nanometer range they may preferably be accumulated in tumor tissue due to the enhanced permeation and retention (EPR) effect. The EPR effect is a hypothesis that has controversial views from different researchers on how to perceive this effect. Although it is believed that EPR is a consequence of the porous vascular endothelium at the tumor site and dysfunctional lymphatic drainage in these tissues [14], some scientists claim that EPR is not present in all types of tumors. Despite the superiority of nanoparticles in passive targeting via EPR effect, desired selectivity to cancer cells and efficient chemotherapy could not be achieved. It was recently suggested that EPR effect may have some limitations due to the absence of blood vessels in or near tumors [15,16]. On the other hand, density of tumor cells makes it difficult to penetrate into the tumor tissue and release the drug bound to the nanoparticles [17]. Another reason for the failure in clinical application is the biological environment which cannot be adequately mimicked in a preclinical/in vitro setting. Naturally, the growth of normal tissue depends on cellular interactions in an environment composed of several growth factors, hormones, and other molecules that are constituents of the extracellular matrix (ECM) [18]. The structure and homeostasis of normal breast parenchyma is maintained by dynamic interactions between breast epithelial cells and their associated stroma. Three-dimensional (3D) cell cultures are suitable to mimic the biological environment at laboratory conditions [19]. 3D spheroids contain an extensive ECM that differs in the relative cell number and assembly from the corresponding conventional monolayer cultures [20]. 3D breast tumor models have an invaluable role in the translation of the tumor biology into breast cancer [21]. 3D in vitro tumor models can mimic the biological multicellular structure of breast cancer to obtain more realistic results.

**Figure 1:**
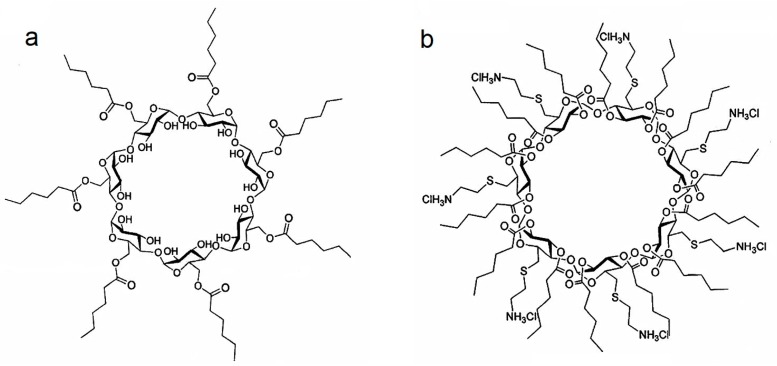
Schematic representation of non-ionic 6OCaproβCD (**a**) and Polycationic PC βCDC6 (**b**).

The aim of this study was to evaluate the effect of PCX:CD inclusion complexes on loading efficiency and the release profile of the drug compared to conventionally prepared nanoparticles. In addition, effects of surface charge of nanoparticles on tumoral penetration were evaluated using in vitro 3D multicellular tumor spheroids. For this purpose, two different amphiphilic CD derivatives were used in this study, namely non-ionic amphiphilic, CD Heptakis (6-O hexanoyl) cyclomaltoheptose (6OCaproβCD) and polycationic amphiphilic CD, (PC βCDC6) as depicted in Figure 1. The safety and efficacy of these amphiphilic CDs were assessed in conventional cell culture methods previously reported by our group [22,23].

## 2. Results and Discussion

### 2.1. Characterization of Paclitaxel:Cyclodextrin (PCX:CD) Inclusion Complexes

**Figure 2:**
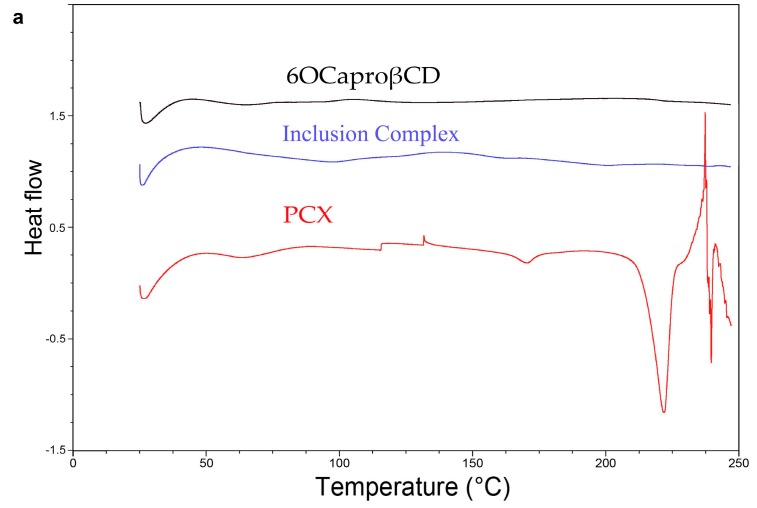

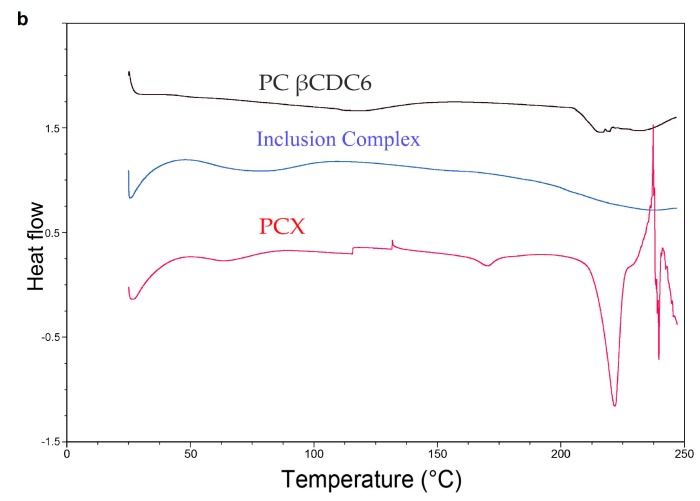
Differential Scanning Calorimetry (DSC) thermograms of Paclitaxel (PCX), 6OCaproβCD, PCX:6OCaproβCD inclusion complex (**a**) PC βCDC6 and PCX:PC βCDC6 inclusion complex (**b**).

Differential Scanning Calorimetry (DSC) thermograms of lyophilized PCX, lyophilized 6OCaproβCD, lyophilized PC βCDC6, lyophilized PCX:6OCaproβCD inclusion complex and lyophilized PCX:PC βCDC6 inclusion complex are shown in Figure 2a,b. As seen in the DSC thermograms, endothermic melting peak is present at 221 °C for PCX, which corresponds to values found in literature [24]. The PCX:6OCaproβCD and PCX:PC βCDC6 inclusion complexes did not show this melting endotherm, suggesting the absence of free PCX crystal in the inclusion complexes. The absence of the PCX melting peak in the CD inclusion complexes confirmed that the complexed PCX possibly is amorphous state [25]. All samples were lyophilized prior to DSC analysis to evaluate the effect of the freeze-drying process on the structure of the drug, the CDs and the complexes. 

**Figure 3:**
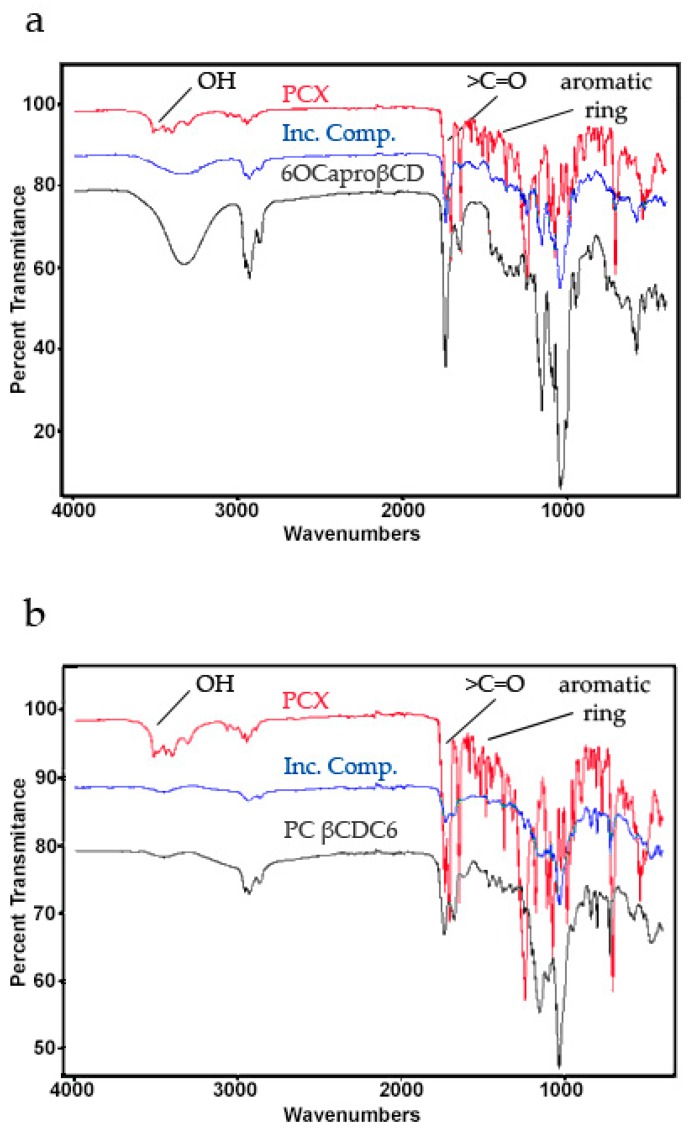
Fourier Transform Infrared Spectroscopy (FT-IR) spectra of PCX, 6OCaproβCD, PCX: 6OCaproβCD inclusion complex (**a**) and PC βCDC6, PCX:PC βCDC6 inclusion complex (**b**).

Fourier Transform Infrared Spectroscopy (FT-IR) spectra of lyophilized PCX, lyophilized 6OCaproβCD, lyophilized PC βCDC6, lyophilized PCX:6OCaproβCD inclusion complex and lyophilized PCX:PC βCDC6 inclusion complex are shown in Figure 3. The spectral analysis indicates an increment in the –OH group for 6OCaproβCD and a reduction for PC βCDC6 inclusion complex. The epoxy group and C–O–C stretching show significant changes. It has been reported that intermolecular hydrogen bonds are formed between the C=O and NH groups in the paclitaxel structure and that the difference observed in the carbonyl region in the Infrared (IR) spectrum indicates a change in these hydrogen bonds [26]. In addition, the significant change observed for the C=O group of PCX suggests that the C=O group of PCX is involved in the CD inclusion complexes [27]. 

**Figure 4:**
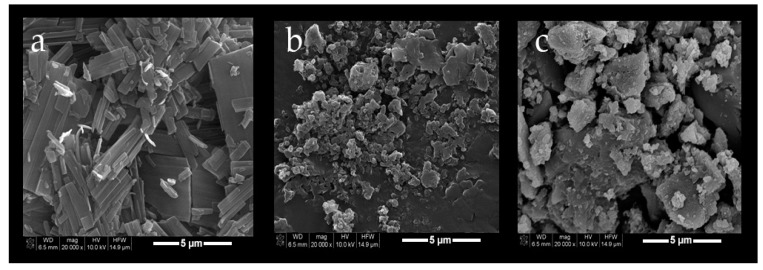
Scanning electron microscopy (SEM) photomicrographs of PCX (**a**); PCX:6OCaproβCD inclusion complex (**b**) and PCX:PC βCDC6 inclusion complex (**c**).

Scanning electron microscopy (SEM) photomicrographs were taken for PCX, PCX:6OCaproβCD inclusion complex and PCX:PC βCDC6 inclusion complex to observe that the typical needle-like structures of dehydrated PCX crystals do not exist in the complexes. Figure 4 represents the SEM photomicrographs of the PCX and PCX:CD inclusion complexes. As seen in the SEM data, no PCX crystals were detected in inclusion complex samples. In previous studies, it was reported that non-ionic 6OCaproβCD and PC βCDC6 can form inclusion complexes with PCX [28]. Our results confirm that PCX is completely included in the hydrophobic CD cavity.

### 2.2. Characterization of Blank or PCX Loaded Nanoparticles 

Table 1 shows the mean particle size, polydispersity index (PDI), and zeta potential values of blank and PCX loaded amphiphilic CD nanoparticles. 

The mean diameter of blank or PCX loaded nanoparticles vary in a range of 75 to 113 nm according to the type of CD used and show a narrow distribution. In addition, drug loading did not cause significant changes on the mean diameter of nanoparticles. Particle size of nanoparticulate drug delivery systems play a direct and important role in cellular uptake, systemic circulation, toxicity and stability of nanoparticles [29]. All nanoparticle formulations showed a particle diameter ranging up to 150 nm, suitable to obtain an effective intracellular uptake [30]. A low PDI was shown for all the formulations (<0.25), indicating homogenous nanoparticle populations. The size of the nanoparticles, which were prepared from the inclusion complexes are larger than nanoparticles prepared conventionally. The reason for this is that the high loading leads to an increase in the particle size of both the amphiphilic cavity and the PCX molecules attached to the hydrophobic alkyl chains. It has been reported that nanoparticles prepared using the pre-loading and high loading technique from inclusion complexes have drug molecules completely contained within the cavity and/or hydrophobic interactions with the long alkyl chains [26]. The particle size of the nanoparticles prepared from the inclusion complexes is also related to the increase in the amount of drug loaded into the complexes. The organic phase was mixed for a long time in the aqueous phase to prepare inclusion complexes. The amount of loaded drug between aliphatic chains increases and the orientation of the chains on the CD surface may be affected by the increasing amount of drug. Though 6OCaproβCD yields to negatively-charged nanoparticles, the 6OCaproβCD molecule is neutral as no charged groups are present in the structure at the normal pH window (2–13). This amphiphilic CD derivative has the unique property to form self-organizing nanoparticles without any surfactant or co-solvent. PC βCDC6 has a strong positive surface charge owing to polycationic amino groups. These differences between surface charge of CD nanoparticles allowed us to compare the effect of surface charge on drug loading capacity, stability and anticancer activity in this study.

**Figure 5:**
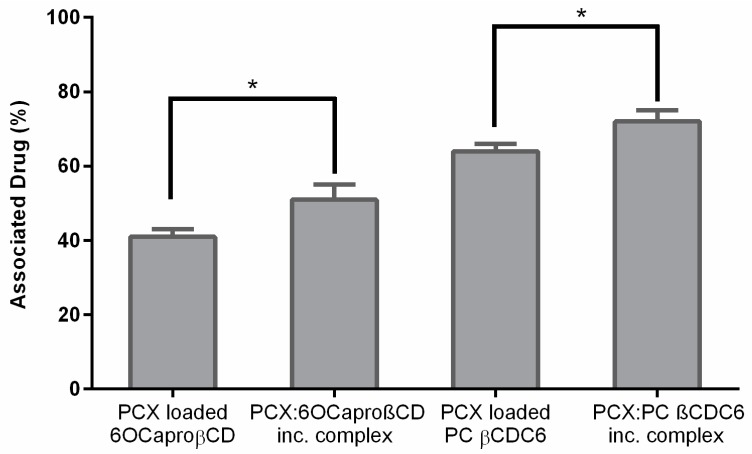
Associated drug (%) of PCX loaded nanoparticles (*n* = 3, ±SD), * *p* < 0.05.

Drug loading values for PCX are seen in Figure 5 in terms of associated drug percentage. It is seen that nanoparticles prepared from PCX:CD inclusion complexes have significantly higher drug loading capacity than conventional nanoparticles (*p* < 0.05). This may be a result of the affinity of this very poorly soluble drug to the inner core of the CD during long term mixing. In addition, PCX with its very poor water solubility and demonstrated affinity to CDs shows a high encapsulation efficiency to CD nanoparticles. In addition, the drug loading ability of CD nanoparticles were related to surface charge of nanoparticles. PCX is negatively charged. The result of electrostatic interaction, PC βCDC6 nanoparticles was higher encapsulation efficacy than the 6OCaproβCD, resulting in 1.2 fold higher loading for PCX PC βCDC6 nanoparticles compared to the negatively charged 6OCaproβCD nanoparticles.

**Figure 6:**
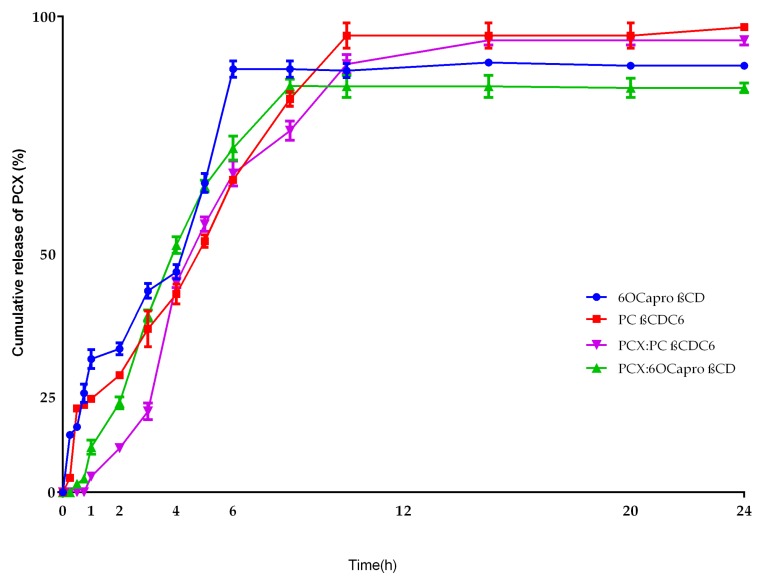
Release profile of PCX from amphiphilic nanoparticles and PCX:CD inclusion complex (*n* = 3, ±SD).

Figure 6 displays the in vitro release profiles of conventional CD nanoparticles and nanoparticles prepared from PCX:CD inclusion complexes. PCX is released over a period of 5 h from non-ionic 6OCaproβCD nanoparticles and 10 h from PC βCDC6 nanoparticles. A burst release of PCX is observed in the first 30 min for conventionally prepared nanoparticle formulations. By contrast, nanoparticles prepared from PCX:CD inclusion complexes liberate the drug at a considerably slower release rate with complete release achieved after 10 h for PCX:6OCaproβCD nanoparticles and 20 h for PCX:PC βCDC6. The amount of PCX is 0.08 mg for 6OCaproβCD nanoparticles and 0.1 mg for PC βCDC6 nanoparticles. Total PCX amount is higher in nanoparticles prepared from inclusion complex; 0.32 mg for PCX:6OCaproβCD nanoparticles and 0.33 mg for PCX:PC βCDC6 nanoparticles.

### 2.3. Cell Culture Studies

#### 2.3.1. Determination IC_50_ of PCX on Co-Culture

**Figure 7:**
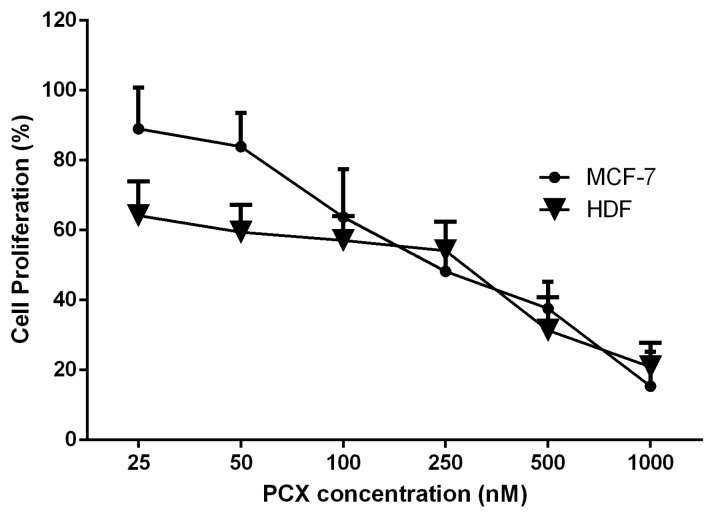
Antiproliferative effect of PCX on MCF-7 human breast cancer cells and HDF human dermal fibroblast cells (*n* = 6, ±SD).

According to the results of antiproliferative studies (Figure 7), IC_50_ values for PCX were determined as 211.3 ± 4.3 nM for MCF-7 human breast cancer cell line and 292.1 ± 5.2 nM for HDF human dermal fibroblast cell. Co-culture studies were carried out with two different cell lines and taking into consideration the IC_50_ results, it was decided to use 200 nM as PCX concentration for all cell culture studies.

In order to examine the antiproliferative effect of PCX on co-cultures, cell mixtures with different ratios of MCF-7 to HDF (1:0, 1:1, 1:1, 3:1 and 0:1) were used. Table 2 shows that when MCF-7 were co-cultured with HDF, the cell viability changes significantly (*p* < 0.05) according to the cell ratio.

The cell viability was 48% in MCF-7 monoculture. In addition, cell viability was higher in the co-cultured groups with higher numbers of fibroblast cells. On the contrary, when MCF-7 cells were co-cultured with equal number of HDF, cell viability decreased, significantly. As shown in Table 2, the IC_50_ value of PCX for HDF cells was found to be higher than for MCF-7 cells. It is also known that fibroblast cells found in tumor stroma play important roles in tumor differentiation, tumor metastasis, and resistance to the anticancer drug [31,32]. 

#### 2.3.2. Determination of Anticancer Activity of Blank or PCX Loaded Nanoparticles on Co-Culture Model

**Figure 8:**
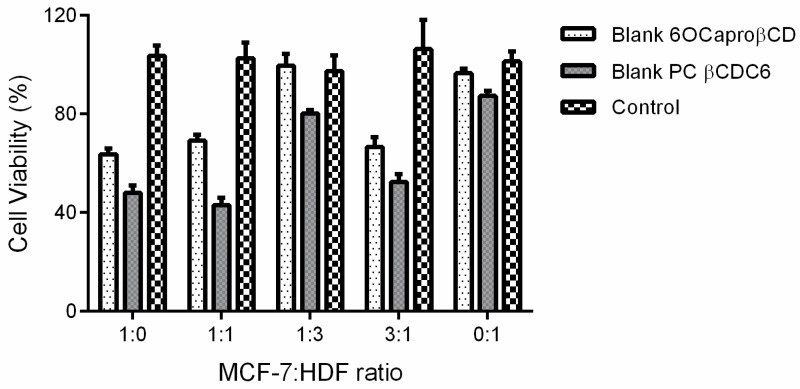
Antiproliferative effect of blank amphiphilic cyclodextrin (CD) nanoparticles on MCF-7: HDF co-culture (*n* = 6, ±SD).

The antiproliferative effect of blank non-ionic and polycationic amphiphilic CD derivatives was determined using the MCF-7: HDF co-culture model. According to results shown in Figure 8, non-ionic and polycationic CD nanoparticles decreased cell viability in groups containing cells at different rates significantly (*p* < 0.05), with the exception of the fibroblast rich group.

Cell viability is reduced in groups with a higher proportion of cancer cells. These results are correlated to the fact that cancer cells are more sensitive than healthy cells to 6OCaproβCD and PC βCDC6 nanoparticles. Our previous studies reported that non-ionic 6OCaproβCD and polycationic βCDC6 nanoparticles induced apoptosis through the mitochondrial pathway targeted to cholesterol microdomains in the cancer cell membrane [23]. Moreover, it was reported that the presence of lipid rafts and especially the concentration of cholesterol are enhanced in several cancer cell membranes [33]. The increased cholesterol level in the cancer cell membrane and the selective affinity of the CDs to cholesterol may be the fact that the blank amphiphilic CD nanoparticles have a higher effect on viability of cancer cells than on healthy cells.

**Figure 9:**
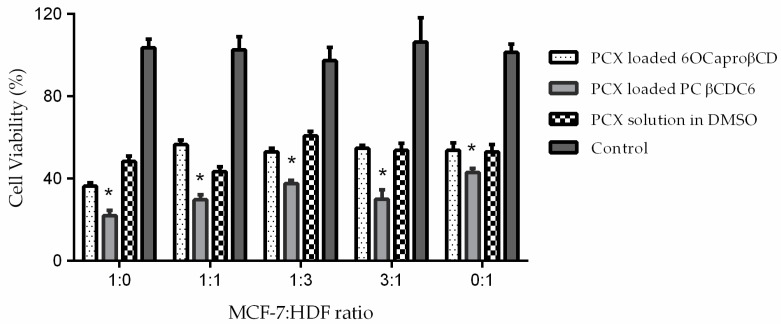
Antiproliferative effect of PCX loaded amphiphilic CD nanoparticles on MCF-7: HDF co-culture (*n* = 6, ±SD) * *p* < 0.05 compared with PCX solution.

The PCX loaded 6OCaproβCD and PC βCDC6 nanoparticles have anticancer activity at least equivalent to the PCX solution in all groups (Figure 9). Especially PC βCDC6 nanoparticles decrease cell viability significantly more than PCX solution, suggesting stronger cellular uptake for the positively charged nanoparticles.

#### 2.3.3. Determination of Antitumoral Activity of PCX Loaded Nanoparticles on 3-Dimensional (3D) Multicellular Tumor Spheroid (MCTS) Cell Culture

**Figure 10:**
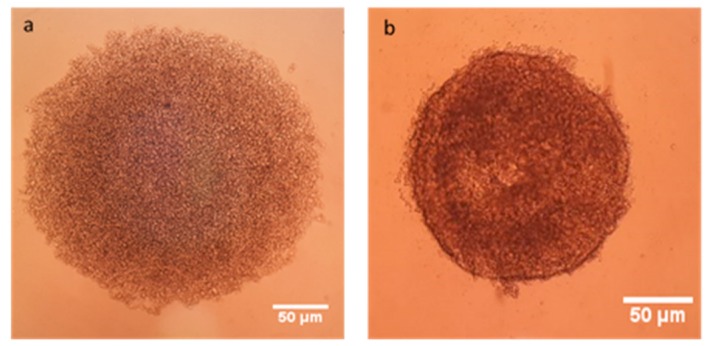
MCF-7 cells after centrifugation (**a**) and 3D MCF-7 spheroid after 7 days (**b**).

3D Multicellular Tumor Spheroid (MCTS) have 3% Matrigel containing cell suspension at different ratio of MCF-7 and HDF cells are given in Figure 10. After centrifugation, cells were collected in the middle of well and after 24 h spheroids were as shown in Figure 10b. There was only one spheroid in each well.

**Figure 11:**
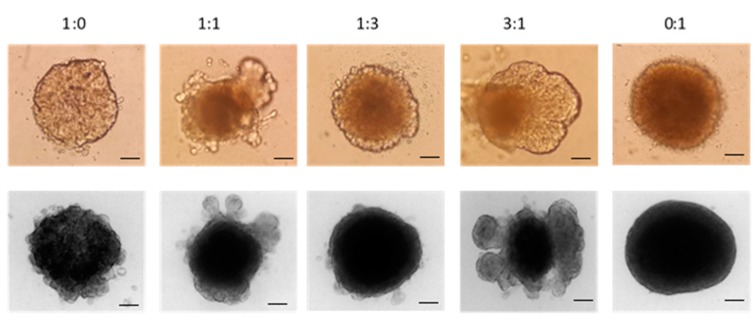
3-Dimensional (3D) spheroids including different ratio MCF-7: HDF cell mixture. The light microscope images (top row) and brightfield microscope images (bottom row). Original magnification: 10×. Scale bar is 50 µm.

Figure 11 shows that the number of fibroblast cells affects the morphology of breast cancer nodules, as higher numbers of fibroblasts in the mixture cause more rigid and smoothly shaped spheroids. On the contrary, spheroids tend to spread out in spheroids in which the cancer cell is more than or equal in cell ratio.

**Figure 12:**
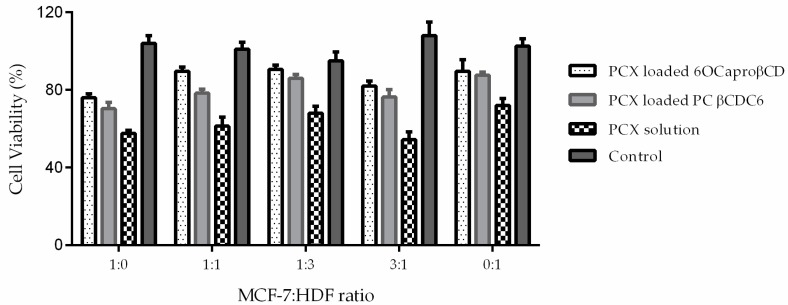
Antitumoral activity of PCX loaded conventionally prepared amphiphilic CD nanoparticles on 3D tumor spheroids (*n* = 6, ±SD).

The antitumoral activity of PCX loaded nanoparticles was evaluated on 3D spheroids with Water Soluble Tetrazolium Salt-1 (WST-1) cell proliferation assay 7 days after the spheroids were formed. According to results shown in Figure 12, non-ionic and polycationic CD nanoparticles decreased cell proliferation to approximately 80% in the spheroid multicellular tumor model.

**Figure 13:**
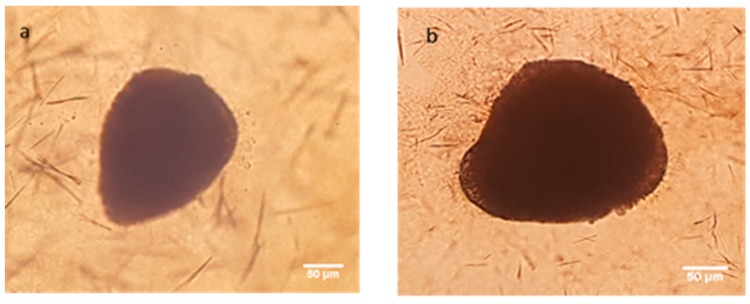
PCX loaded 6OCaproβCD (**a**) and PC βCDC6 nanoparticles (**b**) on MCF-7: HDF spheroids. PCX needle-like crystals observed in both photomicrographs.

In addition, when spheroids were examined after 24 h microscopically, PCX crystals were observed in PCX loaded 6OCaproβCD and PC βCDC6 nanoparticles (Figure 13). PCX was released within 5 and 10 h from non-ionic and polycationic nanoparticles, respectively (Figure 6). Amphiphilic CD uptake from 2D monolayer cells is easily completed within a short time period. However, it takes more time to uptake nanoparticles from 3D multilayer cells, with PCX already released outside the cell before the nanoparticles were taken up by spheroids. Therefore, the conventional PCX loaded nanoparticles had no antitumoral effect on 3D tumor spheroids.

**Figure 14:**
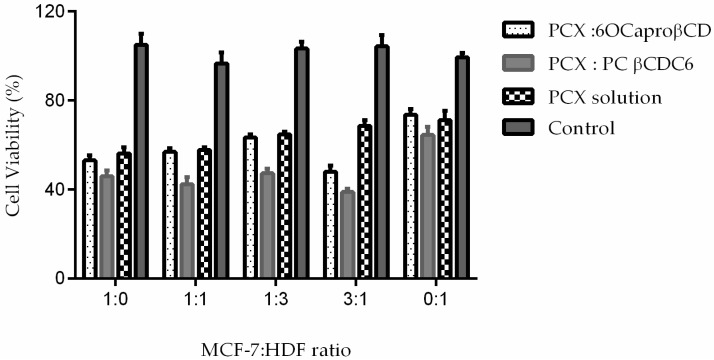
Antitumoral effect of PCX loaded nanoparticles were prepared from inclusion complex on 3D tumor spheroids (*n* = 6, ±SD).

On the other hand, nanoparticles prepared from PCX:CD inclusion complexes behaved differently. Antitumoral activity of nanoparticles prepared from PCX:CD inclusion complex is given by Figure 14. It was clearly shown that after 48 h incubation time nanoparticles prepared from inclusion complexes had higher antitumoral activity against 3D MCTS with different cell ratios. This result is directly related to the PCX release profile from nanoparticles. Each nanoparticle formulation in cell culture contains the PCX at the same concentration. However, PCX released from nanoparticles prepared from inclusion complexes were slower than nanoparticles that were prepared conventionally and therefore more effective in cytotoxicity.

**Figure 15:**
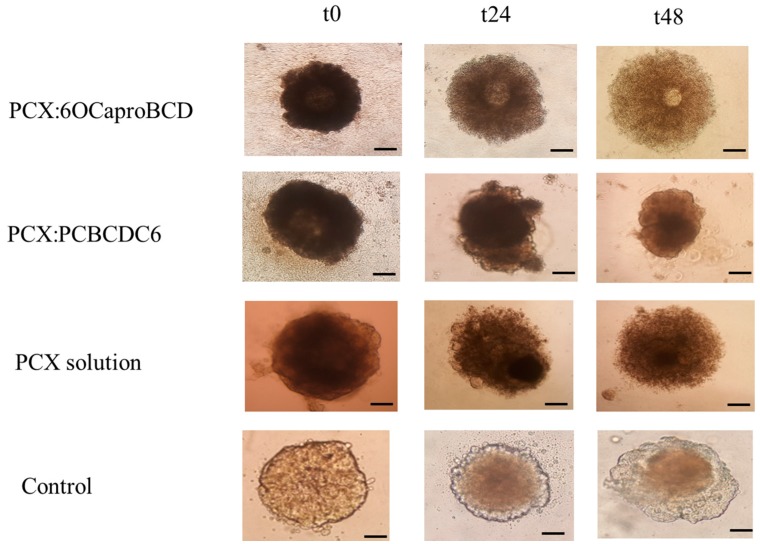
Time dependent microscope image of PCX:CD inclusion complex nanoparticles, PCX dilution and untreated control group. Original magnification: 10×. Scale bar is 50 µm.

After treatment with different nanoparticle formulations, 3D multicellular tumor spheroids were imaged during 48 h. PCX loaded non-ionic amphiphilic CD nanoparticles have similar effects on spheroids when compared to PCX solution. Non-ionic CD nanoparticles and PCX solution cause cell spreading and enlargement of the spheroid. However, polycationic CDs clearly reduced the spheroid size, as seen in Figure 15.

3D in vitro tumor model is bridging 2D cell culture and in vivo animal models in terms of modelling the tumor environment. Tumorigenesis is a biological process controlled by extracellular matrix, cancer cell and stroma. In this biological process, the development and spread of cancer cells depends on many factors, such as growth factors, hormones, and other cells in the extracellular matrix [34,35], thus causing the differences in the results of 2D with 3D cell culture studies. It is thought that 2D cell culture grown on plastic surfaces is not sufficient to mimic tumor conditions in vivo [36,37,38]. On the contrary, 3D tumor spheroids have been shown to better mimic tumor microenvironment in vivo compared to a 2D cell culture method [39]. There are several studies that have shown that 2D cell culture method tend to overestimate the activities of chemotherapeutic drugs compared to 3D method [40,41,42] as observed in our findings, as well.

#### 2.3.4. Determination of Penetration Properties of Amphiphilic CD Nanoparticles on 3D Cell Culture

Nile Red loaded non-ionic and polycationic nanoparticles were prepared to determine uptake by 3D tumors. Seven-day spheroids were incubated with Nile red loaded amphiphilic CD nanoparticles for 6 h and after incubation time spheroids were washed 3 times with Phosphate Buffered Saline (PBS) to remove free dye and nanoparticles that were not taken up by spheroids. 3D spheroids were then imaged with fluorescence microscopy. 

**Figure 16:**
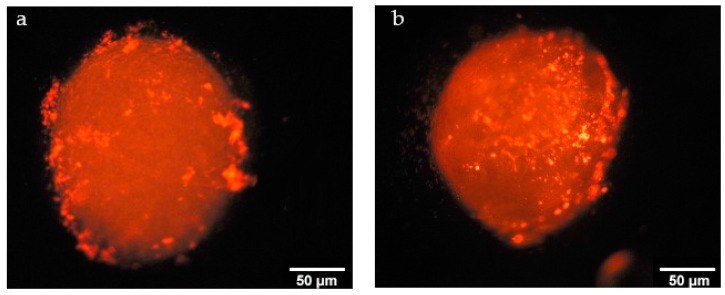
Nile red loaded 6OCaproβCD (**a**) and PC βCDC6 nanoparticles (**b**) on MCF-7: HDF spheroids.

Uptake of Nile Red loaded nanoparticles by nodules is given in Figure 16. Non-ionic and polycationic amphiphilic CDs were taken up by spheroids. Furthermore, polycationic amphiphilic CD nanoparticles can diffuse and penetrate through multiple cell layers. As the cell membrane is negatively charged, the cationic surface charge of nanoparticles enhances the interaction with the cell membrane. Positively charged nanoparticles can bind with negatively charged molecules on the cell membrane and penetrate through bilayers easier and more than non-ionic nanoparticles [23]. In addition, the surface charge of nanoparticles plays an important role in the successive subcellular localization.

## 3. Materials and Methods

Non-ionic amphiphilic CD, Heptakis (6-O hexanoil) cyclomaltoheptose (6OCaproβCD) and Polycationic amphiphilic CD, (PC βCDC6) were synthetized as described previously at the University of Sevilla, Seville, Spain [23]. Paclitaxel (>99% powder, MW: 853.91 g/mol) was purchased from LC Laboratories, (Woburn, MA, USA). All other chemicals used were of analytical grade and obtained from Sigma & Aldrich, Buchs, Switzerland. Ultrapure water was used as obtained from Millipore Simplicity 185 Ultrapure Water System (Millipore, Molsheim, France).

### 3.1. Preparation and Characterization of PCX:CD Inclusion Complexes 

Amphiphilic CDs with different surface charge were used in the study, which were 6OCaproβCD nanoparticles with non-ionic charge and PC βCDC6 nanoparticles with positive charge. 

In order to improve PCX encapsulation efficiency, PCX:CD inclusion complexes were prepared by a co-lyophilization method. To prepare 1:2 PCX:CD inclusion complexes, 5 mg PCX and 27 mg 6OCaproβCD or 5 mg PCX and 33 mg PC βCDC6 were solubilized in ethanol (20 mL) and added dropwise into ultrapure water (40 mL) under magnetic stirring at room temperature during 1 week. The organic phase was evaporated under vacuum at 40 °C. Finally, PCX:CD complexes were lyophilized for 48 h by lyophilization and the resulting powder was characterized by DSC, FT-IR and SEM. 

Differential Scanning Calorimetry (DSC, TA Instruments Q200, New Castle, Delaware, UK) was used to analyze thermal behavior of the model drug, 6OCaproβCD, PC βCDC6 and 1:2 complexes. Each sample (2–4 mg) was heated in a hermetically sealed aluminum pan at a rate of 10 °C/min from 25 to 200 °C under dynamic nitrogen atmosphere. 

Fourier transform infrared (FT-IR) spectra of PCX, 6OCaproβCD, PC βCDC6 and 1:2 complexes were collected between 400 and 4000 cm^−1^ with a Perkin-Elmer BX FT-IR spectrophotometer (Waltham, MA, USA) using previously prepared discs of each sample and potassium bromide containing 0.01 g of sample in 0.1 g of potassium bromide.

The morphology of PCX:CD inclusion complexes and absence of free PCX crystal were evaluated by Scanning Electron Microscope (SEM, FEI Nova™ Nano SEM 430, Hilssboro, OR, USA). Samples were mounted on metal stubs and coated with 100 A thick layer of Gold-Palladium alloy. Then the particles were imaged at 5 to 20 kV.

### 3.2. Preparation and Characterization of Nanoparticles

For PCX loaded nanoparticles drug stock solution (4 mg/20 mL) were prepared in ethanol. In order to prepare drug loaded CD nanoparticles, 2 mg lyophilized inclusion complex or 2 mg amphiphilic CD alone were dissolved in 1 mL PCX stock solution and 1 mL ethanol (2 mL organic phase). Then, the organic phase was dropped into the aqueous phase of ultrapure water (4 mL) under magnetic stirring. The organic phase was evaporated under vacuum and the nanoparticle dispersion was filtered to remove free drug.

The particle size, zeta potential and polydispersity index (PDI) measurements were performed by dynamic light scattering (DLS) (Malvern Zetasizer Nano ZS series, Malvern, Worcestershire, UK) at room temperature using a disposable capillary cell. Each measurement was carried out in triplicate and expressed as mean diameter (nm) ± SD. 

Content of PCX bound in different nanoparticles was quantified by a previously validated High Performance Liquid Chromatography (HPLC) method [5]. Briefly, after the separation of excess amounts of polymers and undissolved and unbound drug by centrifugation at 3500 rpm for 15 min, supernatant containing PCX loaded nanoparticle suspension was freeze-dried for 24 h in order to obtain drug loaded nanoparticles in powder form. Resulting powder was weighed and dissolved in 2 mL mixture of acetonitrile. The experimental PCX loading was quantified using the peak area of each nanoparticle formulation. Drug loading was expressed in terms of associated drug percentage and entrapment efficiency. Associated drug percentage (1) was calculated as follows:

(1) $$\mathrm{Associated}\;\mathrm{Drug}\;\left(\%\right)\;=\;\frac{\;\mathrm{Experimental}\;\mathrm{Drug}\;\mathrm{Loading}}{\mathrm{Theoretical}\;\mathrm{Drug}\;\mathrm{Loading}}\;\times \;100$$

The in vitro release profiles of PCX from nanoparticle formulations were determined at 37 °C using dialysis sacs (molecular weight cut-off value of 100,000 Da) under sink conditions. Briefly, 2 mL of nanoparticle dispersion was put in a dialysis bag, which was placed in 50 mL of phosphate buffer solution (PBS) containing 0.1% *v*/*v* Tween 80 at pH 7.4 to mimic physiological conditions. The system was placed in a shaking water bath at 37 °C with an agitation speed of 100 rpm. Samples were taken from the medium at specific time intervals and replaced with fresh PBS at same volume and temperature. PCX concentrations in samples were determined by HPLC. The measurement of PCX was performed at 227.4 nm. The mobile phase was acetonitrile:water (70:30 *v*/*v*) and the flow rate was set at 1.0 mL/min. The cumulative percentage of drug released for each time point was calculated as a percentage of the total drug incorporated into the nanoparticles.

### 3.3. Cell Culture Studies

In this study, the MCF-7 human breast cancer cell line (ATCC^®^ HTB-22™) and HDF human dermal fibroblast cells were used in all cell culture studies. Dulbecco’s Modified Eagle Medium (DMEM) (Gibco 41965, Life Technologies, Paisley, UK), supplemented with 10% (*v*/*v*) fetal bovine serum (Gibco 10270, Life Technologies, UK), 1% penicillin/streptomycin (15140, Life Technologies, Rochester, NY, USA), 1% (*v*/*v*) l-glutamine (25030, Life Technologies, NY, USA) and 1% (*v*/*v*) Na-pyruvate (11360, Life Technologies, UK) was used for all cell culture studies.

#### 3.1.1. Determination IC_50_ of PCX on Co-Culture

In order to determine IC_50_ values of PCX, MCF-7 and HDF cells were grown in 75 cm^2^ cell flask separately. After the cells reach confluency, both cell lines were harvested using 2 mL trypsin/ethylenediaminetetraacetic acid (EDTA) (5X) solution and cells were stained with trypan blue for counting. Then cells were seeded at 1:0, 1:1, 1:3, 3:1 and 0:1 MCF-7 to HDF ratio in 96-well round bottom cell culture plate with at an initial seeding density of 1 × 10^4^ cells per well in DMEM (200 µL) and allowed to attach overnight. Then media was replaced with different dilutions of PCX stock solution (4 mg/20 mL) in DMEM. Final concentrations of PCX dilutions were 25, 50, 100, 250, 500 and 1 µM, respectively. After 48 h incubation time, cell viability was determined by WST-1 assay. For this purpose, 10 µL WST-1 reagent was added into each well and cell viability was determined with a microplate reader spectroscopically at a wavelength of *λ* = 450 nm. 

#### 3.3.2. Determination of Anticancer Activity of Blank and PCX Loaded Nanoparticles on Co-Culture Model

In order to determine the anticancer activity in co-cultures for the blank CD nanoparticles, drug loaded nanoparticles prepared conventionally or from the inclusion complex, the same protocol was applied for 96-well round bottom cell culture plates. Blank and PCX loaded nanoparticle formulations were diluted with complete DMEM according to the results of the IC_50_ study. Then media was replaced with blank or PCX loaded CD nanoparticle in DMEM. The cells were incubated for 48 h and standard WST-1 protocol was applied to determine cell viability.

#### 3.3.3. Determination of Antitumoral Activity of PCX Loaded Nanoparticles on 3D Cell Culture

The scaffold based method was used for in vitro 3D multicellular tumor spheroid (MCTS) studies, which was described previously by Babic et al. [43]. For this purpose, first 96-well round bottom plates were coated with poly(2-hydroxyethyl methacrylate) (poly-HEMA) (P3932, Sigma, St. Louis, MO, USA) to obtain a low attachment surface. 1.2 g of poly-HEMA was dissolved in 40 mL 95% ethanol and 50 µL of this solution were dispensed into each well of the round bottom plate under sterile conditions. Plates were kept under laminar flow to evaporate solvent for at least 24 h. After this evaporation, plates were covered with lids. 

MCF-7 and HDF cells were cultured in flask separately. After trypsinization, mixed cell types of 1 × 10^4^ cells/mL at different ratios were prepared and 3% Matrigel^®^ Basement Membrane Matrix (356234, Corning, Corning, NY, USA) of total volume was added. 200 µL of cell suspension was dispensed into each poly-HEMA coated well and the plate centrifuged at 1000 rpm for 10 min. Media was changed every 2 days by replacing 100 µL fresh media. The spheroid formation was examined microscopically. After 4 days DMEM was replaced with blank or PCX loaded nanoparticles and 48 h later cell viability was determined by WST-1 assay.

#### 3.3.4. Determination of Tumoral Penetration Properties of Amphiphilic CD Nanoparticles on 3D Cell Culture

In order to observe uptake and penetration properties of non-ionic and polycationic CD nanoparticles on the 3D tumor model, Nile Red loaded nanoparticles were prepared. For their preparation, a stock solution of Nile Red in ethanol (1 mg/10 mL) was prepared. From this stock solution, 100 µL was withdrawn and added to an organic phase including 900 µL ethanol and 1 mg CD. This organic phase was added dropwise to an aqueous phase (2 mL) under magnetic stirring. Organic solvent was evaporated under vacuum at 40 °C. 

Seven days after the spheroids were prepared, 100 µL DMEM was replaced with DMEM including Nile red loaded nanoparticles. After 6 h incubation time, media was removed and spheroids were washed 3 times with PBS to remove free nanoparticles and further imaged with a fluorescence microscope. 

#### 3.3.5. Statistical Analysis

All statistical analyses were performed by Student’s *t*-test using GraphPad Prism version 6 (San Diego, CA, USA). *p* < 0.05 was considered to denote a statistically significant difference.

## 4. Conclusions

In this study, anticancer activity of PCX loaded and blank amphiphilic cyclodextrin nanoparticles with different surface charge were evaluated by MCF-7 alone, HDF alone and MCF-7: HDF co-culture cell studies. In addition, 3D multicellular spheroids were prepared to mimic the tumor microenvironment in vivo. Antitumoral activity and intratumoral penetration properties of PCX loaded amphiphilic cyclodextrin nanoparticles were observed on breast cancer nodules. PCX loaded cyclodextrin nanospheres were found to have higher anticancer effect when compared to the PCX solution against MCF-7 cells on 2D cell culture and co-culture. In addition, 3D spheroid studies showed altered drug response and cell morphology compared with conventional cell culture studies. Kinetics of drug release from nanoparticles play an important role in the antitumoral activity of nano-sized drug delivery systems. The differences between 2D and 3D cell culture methods must be considered while investigating the efficacy of drug delivery systems and optimizing nanoparticles/nanomedicines to be used in vivo studies.

## Figures and Tables

**Table 1:** Mean particle size, polydispersity index (PDI) and Zeta potential of blank or Paclitaxel (PCX) loaded nanoparticles (*n* = 3, ±standard deviation (SD)).

Nanoparticle Formulations	Particle Size (nm) ± SD	PDI ± SD	Zeta Potential (mV) ± SD
Blank 6OCaproβCD	103 ± 1	0.13 ± 0.02	−24 ± 0.3
Blank PC βCDC6	75 ± 2	0.16 ± 0.02	+61 ± 1.4
PCX loaded 6OCaproβCD	113 ± 4	0.22 ± 1	−29 ± 2
PCX loaded PC βCDC6	82 ± 2	0.24 ± 5	+62 ± 1
PCX:6OCaproβCD inclusion complex	135 ± 2	0.13 ± 0.04	−31 ± 3
PCX:PC βCDC6 inclusion complex	120 ± 4	0.15 ± 0.2	+59 ± 2

**Table 2:** Antiproliferative effect of 200 nM PCX solution on human breast cancer cell: human dermal fibrolast cell (MCF-7: HDF) co-culture (*n* = 6, ±SD) * *p* < 0.05 compared with group 1:0 (MCF-7 alone).

MCF-7: HDF Ratio	Cell Viability %
1:0 (MCF-7 alone)	48.4 ± 0.8
1:1	40.2 ± 0.9 *
1:3	62.1 ± 1.5 *
3:1	54.8 ± 1.4 *
0:1 (HDF alone)	51.3 ± 2.1
